# A Bioequivalence Study Comparing Two Pomalidomide Hard Capsule Formulations in Healthy Chilean Subjects

**DOI:** 10.3390/ph19071089

**Published:** 2026-07-15

**Authors:** Marcelo Gomes Davanço, Thaís Pereira Vespasiano, Jessé Moisan, Oscar Gonzalez, Milesa Sarmiento, Mélanie Groleau

**Affiliations:** 1 Medical & Clinical Affairs Department, United Medical Ltd. (A Knight Therapeutics Company), Sao Paulo 04085-001, SP, Brazil; 2 Medical & Clinical Affairs Department, Knight Therapeutics Inc., Montreal, QC H4M 2P2, Canada; 3 R&D Department, Laboratorio LKM S.A. (A Knight Therapeutics Company), Buenos Aires C1179, Argentina

**Keywords:** pomalidomide, bioequivalence, bioavailability, pharmacokinetics, generics, multiple myeloma

## Abstract

**Background:** Multiple myeloma (MM) is a mature B-cell disorder characterized by the excessive production of monoclonal immunoglobulins. It is the second most common hematological cancer and predominantly affects older adults. In 2022, there were approximately 188,000 MM new cases worldwide. The disease is associated with a relapsing–refractory course. First-line therapies are often insufficient, making additional treatment options necessary. For patients refractory to lenalidomide and proteasome inhibitors (bortezomib and/or carfilzomib), pomalidomide combined with dexamethasone and an additional active agent can be used as a therapeutic strategy. **Objective**: This study was conducted to evaluate the bioequivalence and tolerability of two pomalidomide hard capsule formulations to support regulatory approval of a branded generic product in Latin American countries. **Methods**: An open-label, randomized, single-dose, two-treatment, two-sequence, two-period crossover study was conducted in healthy male subjects. Participants received a single oral dose of the test product, Xetrane^®^ 4 mg hard capsule (Laboratório LKM S.A., Argentina), and the reference product, Imnovid^®^ 4 mg hard capsule (Celgene International Sàrl), with a 7-day washout period. Blood samples were collected over 48 h post-dose. Plasma pomalidomide concentrations were determined using a validated LC-MS/MS method, and pharmacokinetic parameters were estimated using non-compartmental analysis. **Findings**: Thirty-four subjects were enrolled, and 29 completed the study. Geometric mean ratios (90% confidence intervals) for Cmax and AUC_0–t_ were 106.24% (100.77–112.00) and 94.67% (91.61–97.82), respectively. Both formulations were well tolerated. Bioequivalence between Xetrane^®^ and Imnovid^®^ was demonstrated in accordance with regulatory criteria. NCT07694011.

## 1. Introduction

Multiple myeloma (MM) is an aggressive malignant neoplasm of plasma cells derived from mature B cells, characterized by their unregulated clonal proliferation within the bone marrow [1]. This accumulation leads to the excessive production and release of M protein, an anomalous monoclonal immunoglobulin, or its fragments [2]. The burden of clonal plasma cells and elevated monoclonal immunoglobulin levels in the bone marrow can result in bone destruction and marrow failure [3,4,5]. Clinically, MM may manifest with osteolytic lesions, hypercalcemia, renal insufficiency, suppression of hematopoiesis (anemia) and humoral immunity [2,6]. Usually, the incidence increases with age; most cases are diagnosed in individuals over 65 years old [1,2]. It accounts for approximately 15 percent of all hematologic malignancies and 20 percent of deaths from this group of neoplasms [1].

MM has a complex, multistep pathogenesis involving genetic alterations and microenvironmental interactions; however, its precise initiating causes are not fully understood [1]. According to several studies, there is strong evidence that environmental exposures to certain chemicals, pesticides and radiation, obesity, chronic inflammation and genetic events may act as risk factors for the development of the disease [1,4]. Furthermore, MM progresses through well-recognized precursor stages before the onset of symptoms. The first stage involves the monoclonal gammopathy of undetermined significance (MGUS), where biomarkers such as M protein or clonal B plasma cells remain below diagnostic thresholds. The intermediate stage is smoldering multiple myeloma (SMM), an asymptomatic precursor to multiple myeloma. While patients remain free of myeloma-defining events, some may exhibit high-risk features. High-risk SMM is commonly defined by the presence of ≥2 of the following parameters: bone marrow plasma cells ≥ 20%, serum M-protein ≥ 2 g/dL (20 g/L), and a serum free light chain (FLC) ratio > 20. By definition, SMM lacks myeloma-defining events, including CRAB features or SLiM biomarkers such as ≥60% marrow plasma cells, FLC ratio ≥ 100, or more than one focal lesion on MRI. However, limited abnormalities, such as a single focal lesion on MRI, may still be observed in SMM. Disease progression correlates with biomarker levels at diagnosis, and genetic events become progressively more pronounced as the disease approaches its final stage, multiple myeloma (MM) [4,7,8].

Pomalidomide possesses potent immunomodulatory and significant antimyeloma properties [5]. The FDA has approved pomalidomide for patients with MM who have received at least 2 prior therapies including lenalidomide and bortezomib and have demonstrated disease progression on or within 60 days of completion of the last therapy. Similarly, the National Comprehensive Cancer Network Guidelines Panel includes pomalidomide plus dexamethasone as a therapeutic option for patients who have received at least two prior therapies, including an immunomodulator agent and a proteasome inhibitor, and have demonstrated disease progression on or within 60 days of completion of the last therapy (category 1) [5]. Recent treatment paradigms increasingly incorporate pomalidomide into triplet regimens, typically in combination with dexamethasone and anti-CD38 monoclonal antibodies or other novel agents, with demonstrated improvements in efficacy compared to doublet therapy, particularly in lenalidomide-refractory disease. Emerging data also support the evaluation of pomalidomide within quadruplet regimens and other multi-agent combinations, including those incorporating bispecific antibodies such as talquetamab. Overall, these developments underscore the continued clinical relevance of pomalidomide as an important component of relapsed/refractory multiple myeloma treatment strategies [5,9,10,11,12]. 

Pomalidomide is currently available as hard capsules for oral administration. Maximum plasma concentration (Cmax) is typically reached between 2 and 3 h after oral administration. The drug exhibits dose-proportional and linear pharmacokinetics over the therapeutic dose range, with systemic exposure increasing proportionally [8]. Pomalidomide is extensively metabolized prior to excretion, and its metabolites are primarily eliminated via the urine. It is eliminated by a variety of routes, such as cytochrome P450 (CYP)-mediated metabolism, non-CYP-dependent hydrolysis, and excretion of unchanged drug. In healthy volunteers, the drug exhibits a mean plasma half-life of approximately 9.5 h, whereas in patients with multiple myeloma the half-life is about 7.5 h. The average apparent clearance after oral administration is about 7–10 L/h [13]. 

Access to oncology medicines in Latin America remains heterogeneous, with specific barriers including delayed reimbursement decisions and/or restricted inclusion in public formularies. In healthcare systems that are predominantly public or mixed, such as those in Chile and Colombia, the availability of additional quality-assured therapeutic options is a key strategy to enhance the sustainability of onco-hematological care programs and to improve patient access to essential treatments [14,15,16]. International and regional health organizations have consistently highlighted the importance of promoting the use of safe, effective, and affordable treatments to address disparities in access to cancer therapies across Latin America [14,15,16,17].

Pomalidomide presents a well-characterized pharmacokinetic profile that allows the demonstration of bioequivalence through comparative pharmacokinetic studies. Bioequivalence studies are designed to demonstrate that different formulations containing the same active pharmaceutical ingredient, when evaluated under identical experimental conditions, exhibit comparable bioavailability in terms of both the rate and extent of absorption. This evaluation involves a comparative analysis of the pharmacokinetic profiles of the test and reference formulations, followed by statistical assessment of relevant pharmacokinetic parameters to establish bioequivalence. The main purpose of the current study was to assess the bioequivalence and tolerability of two pomalidomide formulations to meet the regulatory requirements for the registration of a branded generic product (Xetrane^®^) in Latin American markets.

## 2. Results

### 2.1. Bioequivalence Study

#### 2.1.1. Demographic Data

**Figure 1:**
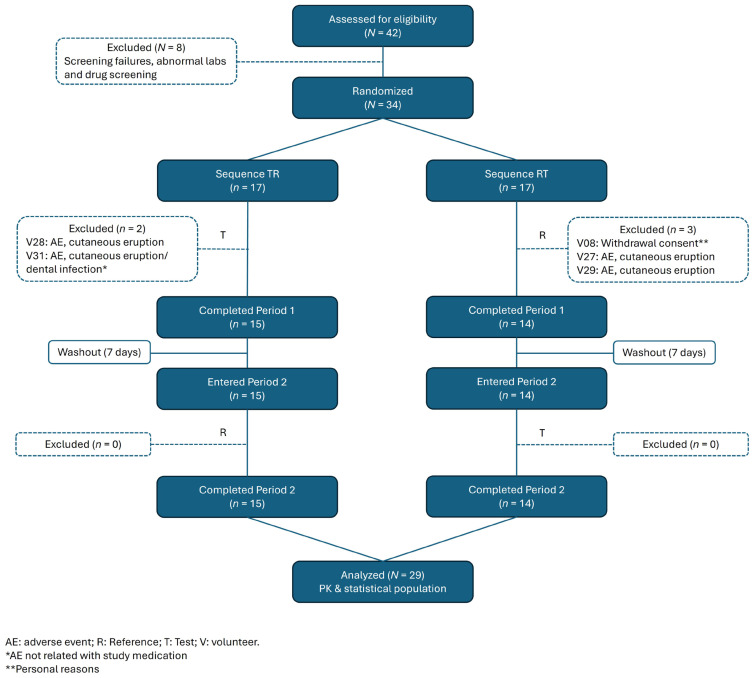
Flowchart of subject disposition in the bioequivalence study.

After the medical history assessment, verification of vital signs, physical examination, electrocardiogram, and routine laboratory tests, all the subjects showed good health conditions and the absence of significant diseases. Thirty-four (34) healthy subjects were enrolled in the study; twenty-nine (29) completed the two periods and were included in the pharmacokinetic and statistical analysis (Figure 1). The demographic characteristics of the study subjects who completed all phases of the study are shown in Table 1.

#### 2.1.2. Bioanalysis

The bioanalytical method was fully validated for all the relevant assays (selectivity, carryover, precision, accuracy, matrix effect, dilution integrity, stability, and calibration curve), and it was confirmed to have adequate selectivity (no interference from blank plasma substances at the retention times of pomalidomide and IS [pomalidomide-d5], an LLOQ of 1.25 ng/ml), no carry-over effects (no pomalidomide was detected in the pre-dose plasma samples of any subject), and a linear calibration curve (1.25 to 100 ng/mL). In terms of precision and accuracy, the method was suitable both when intra-run and inter-run, and the samples were stable at room temperature for up to 4 h in plasma and up to 24.5 h in solution, even after three and six freezing and thawing cycles in a standard freezer (−20 °C), and even after 82 days. These are important results if the samples are to be stored properly before drug concentration analysis. To summarize, all validation parameters were within the pre-defined acceptance criteria according to INVIMA and ISP guidelines [18,19,20,21]. This confirms the validity and robustness of the method for quantifying plasma concentrations of pomalidomide.

#### 2.1.3. Pharmacokinetic Analysis and Bioequivalence Outcome

**Figure 2:**
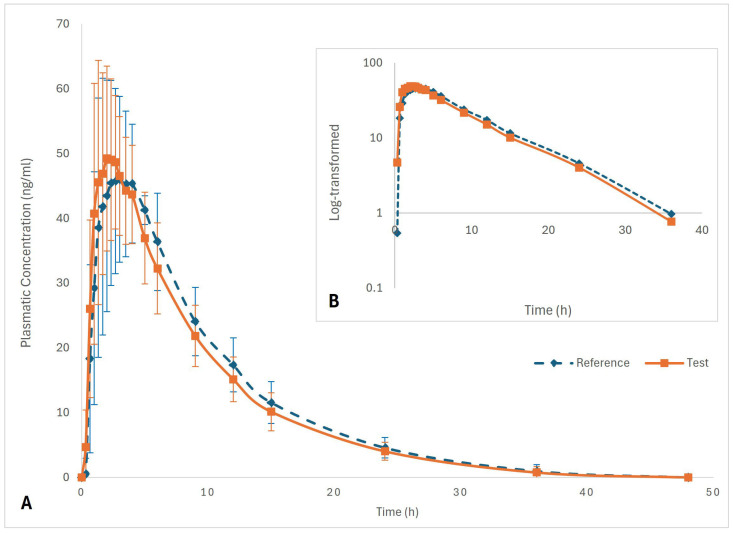
Plasma concentration–time profiles of the test and reference formulations in healthy subjects under fasting conditions. (**A**) Linear scale pharmacokinetic profiles. (**B**) Log-transformed pharmacokinetic profiles. (*N* = 29; mean ± SD).

The mean plasma concentration vs. time curves of pomalidomide (reference and test formulations) are shown in Figure 2. The test and reference products exhibited similar pharmacokinetic profiles, and the sampling duration was sufficient to reliably capture the absorption and elimination phases.

Table 2 describes the pharmacokinetic parameters of pomalidomide for both formulations.

The percentage of AUC extrapolated from the last measurable concentration to infinity (%AUCextrap) was evaluated for all subjects, and values were below 20%, confirming the reliability of total exposure estimates. Predose concentrations were below the lower limit of quantification (LLOQ) for all subjects.

**Figure 3:**
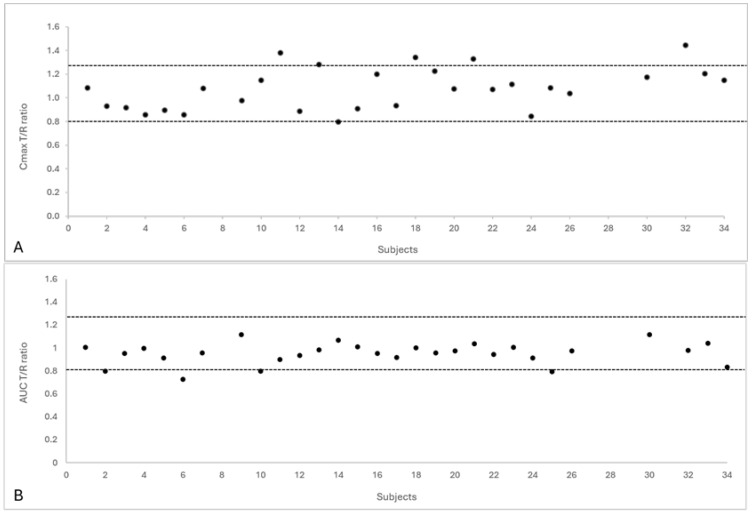
Dispersion of T/R ratio for Cmax (**A**) and AUC_0–t_ (**B**) between study subjects (*N* = 29). The dashed lines represent the bioequivalence limits (0.80–1.25).

The distribution of T/R ratios for Cmax and AUC_0–t_ for the 29 subjects who completed both study periods are presented in Figure 3, and the data shows the distribution is narrow, suggesting low intra-subject variability between formulations for these pharmacokinetic metrics, which is quantitatively confirmed by the within-subject coefficient of variation (CVws) of 11.85% for Cmax and 7.34% for AUC_0–t_ (Table 3).

Table 3 summarizes the pharmacokinetic parameters, including test/reference geometric mean ratios, 90% confidence intervals, within-subject variability (CV_ws_), and statistical power for the bioequivalence assessment of C_max_, AUC_0–t_ and AUC_0–inf_.

The 90% confidence intervals of the test/reference geometric mean ratios for pharmacokinetic metrics were entirely contained within the bioequivalence acceptance limits of 80.00–125.00%.

#### 2.1.4. Safety/Tolerability

Twelve non-serious adverse events were reported during the study (Table 4). Of these, 11 were probably related to the study medication, including pruritus (four cases in the test formulation and three in the reference product) and cutaneous eruptions (two cases in each formulation), which are consistent with the known safety profile of immunomodulatory agents such as pomalidomide [13]. One event, a dental infection, was deemed unlikely to be related to the study medication. All adverse events were mild in intensity, and no serious adverse events were observed throughout the study.

Among the twelve non-serious adverse events reported, five required pharmacological treatment for resolution, including cases of cutaneous eruption on the scalp and dental infection. For the management of cutaneous eruption, treatment included oral desloratadine and topical mometasone applied to the affected area, or oral betamethasone combined with dexchlorpheniramine and topical mometasone. In the case of dental infection, oral azithromycin was prescribed, with paracetamol administered as needed in the presence of fever.

For safety and ethical reasons, the four subjects who experienced cutaneous eruption or dental infection were withdrawn from the study in Period 1. However, all subjects were followed until complete resolution of the adverse events.

Importantly, adverse event-related discontinuations occurred after both formulations (Test and Reference), without evidence of imbalance in tolerability between treatments.

One participant withdrew consent for personal reasons during Period 1. Thus, 29 subjects completed both study periods and were included in the pharmacokinetics analysis set. 

### 2.2. Biowaiver for the Lower Strength

Qualitative and quantitative proportionality between the 3 mg and 4 mg strengths was confirmed, as both formulations contained identical excipients in the same relative proportions and were manufactured using the same process. 

**Figure 4:**
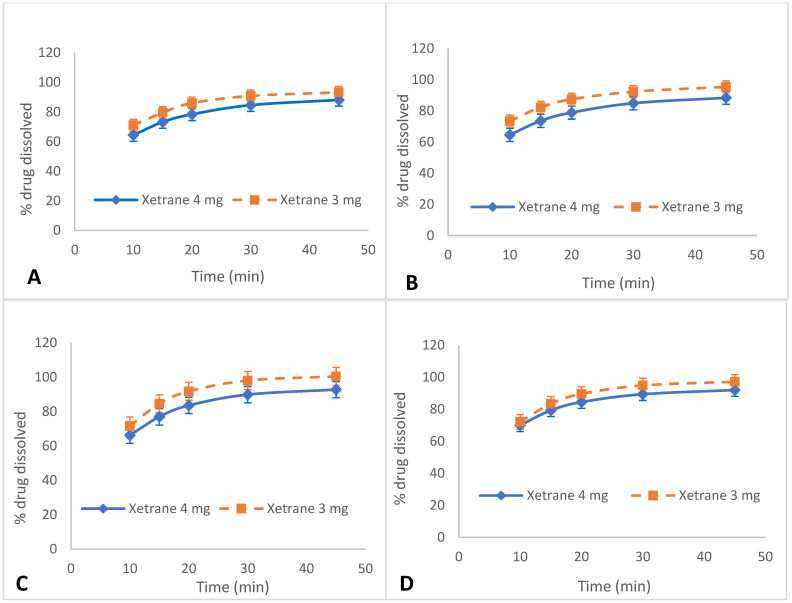
Comparative dissolution profiles of pomalidomide 3 mg and 4 mg hard capsules in different dissolution media: (**A**) quality control medium (0.1 N HCl), (**B**) pH 1.2 (0.05 N HCl), (**C**) pH 4.5 acetate buffer, and (**D**) pH 6.8 phosphate buffer. Results are expressed as mean ± SD (*n* = 12 vessels).

Comparative dissolution profiles of the two strengths were evaluated under four dissolution conditions, including quality control medium (0.1 N HCl), pH 1.2 (0.05 N HCl), pH 4.5 acetate buffer, and pH 6.8 phosphate buffer. As shown in Figure 4, the dissolution profiles of the pomalidomide 3 mg closely overlapped with those of the 4 mg strength in all media tested: (A) quality control medium, (B) pH 1.2, (C) pH 4.5, and (D) pH 6.8. 

The similarity between dissolution profiles was quantitatively assessed using the similarity factor (f_2_). For all dissolution media, the calculated f_2_ values exceeded the acceptance threshold of 50, demonstrating similarity between the dissolution behavior of the lower-strength (3 mg) and higher-strength (4 mg). The f_2_ results for each dissolution condition are summarized in Table 5. Test variability was controlled by ensuring that the coefficient of variation at each time point was within the acceptable limits (≤20% at early time points and ≤10% thereafter). These data support the reliability of the mean profiles used for f_2_ calculation.

These findings confirm that the in vitro performance of the pomalidomide 3 mg hard capsule is comparable to that of the 4 mg strength (bio-batch), which was demonstrated to be bioequivalent to the reference product in vivo. Taken together, the excipient proportionality, the linear pharmacokinetics of pomalidomide and the similar dissolution profiles across all tested media support the scientific justification for waiving an additional bioequivalence study for the 3 mg strength.

## 3. Discussion

FDA recommends a single-dose bioequivalence study, with the highest strength, in healthy subjects and under fasting conditions for the regulatory approval of generic pomalidomide capsule formulations [22]. The current study showed that the test and reference are bioequivalent, and their safety and tolerability were evaluated by administering them to healthy subjects.

The primary pharmacokinetic metrics of the test product were found to be comparable to the reference product, and the 90% confidence intervals of the test-to-reference geometric mean ratios of Cmax and AUC_0–t_ were 100.77–112.00% and 91.61–97.82%, respectively. This is within the pre-defined bioequivalence acceptance range of 80.00–125.00%.

In the present study, mean Cmax values were 59.08 ± 12.07 ng/mL for the test formulation and 55.54 ± 11.10 ng/mL for the reference formulation, while mean AUC_0–t_ values were 487.08 ± 102.32 ng·h/mL and 515.72 ± 112.51 ng·h/mL, respectively. Similarly, total systemic exposure assessed by AUC_0–inf_ showed close agreement between formulations (510.67 ± 101.47 ng·h/mL for test and 539.07 ± 111.51 ng·h/mL for reference). The median Tmax was identical for both products (2 h), indicating comparable absorption rates, while the mean elimination rate constant (Kel) and terminal half-life (t_1/2_) were also similar between formulations (Kel: 0.1131 ± 0.0209 h^−1^ vs. 0.1115 ± 0.0185 h^−1^; t_1/2_: 6.35 ± 1.31 h vs. 6.40 ± 1.21 h for test and reference, respectively). These results confirm that both formulations exhibit comparable absorption, distribution, and elimination characteristics, consistent with the well-described linear pharmacokinetics of pomalidomide reported in the literature. These findings further support the reliability of the results and confirm the adequacy of the study design for the assessment of bioequivalence.

Hoffmann et al. evaluated the absorption, metabolism, and excretion of pomalidomide following a single oral dose in healthy male subjects. They demonstrated that pomalidomide is well absorbed, with a median Tmax of approximately 2.5 to 3 h and a terminal half-life ranging from 8.9 to 11.2 h. In addition, systemic exposure was characterized by mean Cmax values in the range of approximately 40–60 ng/mL and AUC values generally between 400 and 600 ng·h/mL. The parent compound accounted for the majority of circulating drug-related material, with extensive metabolism primarily mediated by CYP1A2 and CYP3A4 [23].

Wang et al. conducted a randomized, open-label, single-dose, two-period, crossover bioequivalence study in healthy male volunteers under fasting and fed conditions, administering pomalidomide 4 mg capsules. Under fasting conditions, mean Cmax values were approximately 50–65 ng/mL, while AUC_0–t_ values were in the range of 450–600 ng·h/mL. The authors concluded that the test and reference formulations had similar pharmacokinetic profiles, with the 90% confidence intervals for the geometric mean ratios of Cmax, AUC_0–t_, and AUC_0–inf_ entirely within the predefined bioequivalence acceptance range of 80.00–125.00%. A delay in Tmax (approximately 2 to 3 h) and a reduction in Cmax (around 20%) were observed under fed conditions; however, the extent of exposure (AUC) remained comparable, indicating no clinically relevant food effect [24].

Similar findings were reported by Liu et al., who evaluated two strengths of pomalidomide (1 mg and 4 mg) in healthy subjects under fasting and fed conditions. In the 4 mg fasting arm, mean Cmax values were generally reported in the range of 50–70 ng/mL, with AUC_0–t_ values approximately between 450 and 650 ng·h/mL. The terminal half-life was around 7–10 h, and tmax was typically observed between 2 and 3 h. In this study, all primary pharmacokinetic parameters met bioequivalence criteria, and the incidence and severity of adverse events were comparable between the generic and reference products, confirming the consistent and predictable pharmacokinetic behavior of pomalidomide across formulations [25]. 

Regarding safety, pomalidomide formulations demonstrated an acceptable and well-characterized safety profile in healthy male subjects, with non-serious adverse events of mainly mild intensity and consistent with the known pharmacological effects of immunomodulatory agents. No new or unexpected safety signals related to systemic exposure were identified in this study, and the safety profile observed was consistent with that reported in the reviewed literature [23,24,25,26]. However, the interpretation of these findings is subject to limitations as the study was conducted as a single-dose trial in healthy male volunteers, which may not fully reflect the safety profile observed under chronic use in the target patient population.

In accordance with the FDA product-specific guidance for pomalidomide capsules [22], additional in vivo bioequivalence studies for lower strengths may be waived, provided that proportional similarity among formulations and comparable in vitro dissolution profiles are demonstrated, supported by the linear pharmacokinetics of pomalidomide. Accordingly, the 3 mg strength was adequately compared with the 4 mg bio-batch through dissolution profile testing in multiple media, demonstrating similarity in performance.

Finally, based on our review of the available published literature, no previous bioequivalence studies of pomalidomide hard capsules conducted in Latin American populations were identified. Therefore, to the best of our knowledge, the present study represents the first report in this population.

## 4. Material and Methods

### 4.1. Drug Products

The test formulation was a pomalidomide 4 mg hard capsule (Xetrane^®^, lot number: L867D, expiry date: August 2025, Knight Therapeutics Inc., Quebec, QC, Canada), manufactured by Laboratorio LKM S.A. (Buenos Aires, Argentina). The reference product was the Imnovid^®^ (pomalidomide) 4 mg hard capsule (lot number: C2643CA, expiry date: August 2027, Bristol Myers Squibb, Princeton, NJ, USA) manufactured by Celgene International Sarl (Boudry, Switzerland). Administration of the drug was carried out in two periods and two groups: 25 January 2025 (Period 1, Group 1), 29 January 2025 (Period 1, Group 2), 01 February 2025 (Period 2, Group 1) and 5 February 2025 (Period 2, Group 2). Both administrations were within the labeled shelf-life of the test and reference products, and the products had been stored under recommended conditions for the duration of the study.

### 4.2. Bioequivalence Study

#### 4.2.1. Ethical Aspects and Good Clinical Practice

The clinical phase of the bioequivalence study was conducted at Domínguez Lab in Chile, while the analytical phase was performed at the Domínguez Lab facility in Argentina. The study protocol was reviewed and approved by the *Comité Ético Científico del Servicio de Salud Metropolitano Oriente de Santiago de Chile* (CEC-SSMO) on 14 January 2025. 

According to Chilean regulatory requirements [18,19], bioequivalence studies intended for regulatory purposes must have their study protocols formally submitted to and approved by the Institute of Public Health (ISP) prior to study initiation. Consequently, a regulatory approval was required from the ISP (*Resolución Exenta* RW n. 38032/24, 21 October 2024).

All subjects provided informed consent before any study procedures were initiated.

The study was conducted in accordance with Good Clinical Practice [27], the ethical guidelines for human research participants as outlined in the Declaration of Helsinki [28], and the applicable regulations from ISP and INVIMA [18,19,20,21]. 

#### 4.2.2. Study Design

The study was a single-center, randomized, single-dose, 2-period, 2-treatment, and 2-sequence crossover design. In this design, gender balance was not applicable, as only male subjects were enrolled due to the known embryo–fetal toxicity of pomalidomide and associated risk mitigation measures, despite the drug being indicated for both male and female patients with multiple myeloma [13].

The subjects were randomly assigned to receive either the test or the reference formulations after a 7-day washout period. A stratified block randomization scheme was applied, using ETCETERA version 3.26^®^ (J.H. Abramson, 1993–2016; WINPEPI), whereby each block (subject) was given the two formulations during different periods, with sequences randomly assigned and balanced to reduce the impact of sequence and period effects.

Subjects fasted for at least 10 h and no more than 16 h overnight prior to drug administration, then received a single oral dose of 4 mg in 240 mL of water and remained fasting for 4 h. To standardize the treatment groups, subjects in both periods followed an identical standard diet.

Twenty blood samples were taken at 0 h (30 minutes prior to drug administration) and 0.33, 0.67, 1.00, 1.33, 1.67, 2.00, 2.33, 2.67, 3.00, 3.50, 4.00, 5.00, 6.00, 9.00, 12.00, 15.00, 24.00, 36.00, 48.00 h post drug administration. The blood samples were centrifuged at 3000 rpm for 5 min at 4 °C, the plasma was separated, placed into tubes, appropriately labeled, and stored at −20 °C until ready for analysis. 

The primary endpoint was a pharmacokinetic comparison of the test and reference formulations based on the maximum plasma drug concentration (Cmax) and area under the curve from time zero to the last quantifiable concentration (AUC_0–t_).

#### 4.2.3. Study Population

Thirty-four healthy male subjects between 18 and 55 years of age with a body mass index (BMI) between 18.5 and 30 kg/m^2^ were enrolled in the study. The inclusion criteria were as follows: subjects had not participated in another clinical trial in the past six months, had not donated blood in the past three months, and had no history of alcohol or drug abuse, were in good health with no clinically significant diseases as determined by medical history, vital signs, physical examination, electrocardiogram (ECG), and routine laboratory tests. Biochemical analyses included liver function parameters (aspartate aminotransferase [AST], alanine aminotransferase [ALT], total bilirubin, and alkaline phosphatase) and renal function parameters (serum creatinine and urea). Only subjects with laboratory values within normal reference ranges or considered not clinically significant by the investigator were enrolled. Individuals presenting clinically relevant abnormalities, particularly those suggestive of hepatic or renal impairment, were excluded.

Subjects’ clinical histories were also reviewed for gastrointestinal disorders, hypersensitivity to pomalidomide or any excipients of the formulation, and the use of concomitant medications that could potentially interfere with pomalidomide pharmacokinetics.

To ensure compliance with the study protocol, subjects remained confined at the clinical facility for approximately 36 h, during which all procedures related to blood sample collection, adherence assessment, and safety monitoring were performed.

Moreover, subjects were fully informed about the purpose, nature, risks, and potential harms of the study, and signed written informed consent indicating their willingness and ability to comply with all study procedures.

#### 4.2.4. Bioanalytical Method

Pomalidomide was extracted from a 200 µL aliquot of human plasma by protein precipitation using acetonitrile, with pomalidomide-d5 (deuterium-labeled analog) used as the internal standard. 

Chromatographic separation was performed on a Shimadzu LC-20AD XR system equipped with an autosampler (SIL-20A XR) and column oven (CTO-20A) (Shimadzu Corporation, Kyoto, Japan), coupled to an AB Sciex 5500 triple-quadrupole mass spectrometer (AB Sciex/SCIEX, Concord, ON, Canada). An aliquot of 5 µL of each processed sample was injected into the LC-MS/MS system. 

The mobile phase consisted of acetonitrile (mobile phase A) and 10 mM ammonium acetate (mobile phase B), delivered by a binary pumping system under validated conditions. Detection was carried out using positive electrospray ionization in multiple-reaction monitoring mode. 

The validated bioanalytical method fulfilled all regulatory requirements, including selectivity, carryover, linearity, precision, accuracy, matrix effect, dilution integrity, and stability evaluations. The method was linear over the concentration range of 1.25 to 100 ng/mL, using nine non-zero calibration levels plus blank and zero samples. A weighted (1/x) linear regression model was applied, with correlation coefficients consistently exceeding 0.999. 

The lower limit of quantification (LLOQ) was established at 1.25 ng/mL, with acceptable precision and accuracy. Intra- and inter-run precision (expressed as coefficient of variation, CV%) and accuracy were within ±15% for all quality control (QC) levels and within ±20% at the LLOQ.

Selectivity was demonstrated across six independent plasma sources (normal, lipemic, and hemolyzed), showing no significant interference at the retention times of the analyte or internal standard. Carry-over was not observed, as responses in blank samples were below 20% of the LLOQ for the analyte and below 5% for the internal standard.

Matrix effects were evaluated using multiple plasma sources and were found to be negligible, with normalized matrix factor variability (CV) below 15%, confirming the absence of significant ion suppression or enhancement. Dilution integrity was established for samples exceeding the upper limit of quantification, demonstrating acceptable accuracy and precision within predefined acceptance criteria.

Stability of pomalidomide was confirmed under all relevant conditions, including short-term stability at room temperature (up to 24.5 h), post-preparative stability in the autosampler (up to 25.5 h), freeze–thaw stability after three cycles, and long-term stability under frozen conditions (at least 80 days at −20 °C). No significant degradation was observed under these conditions.

The bioanalytical results minimized inter-assay variability because all samples from the same subject were analyzed within the same chromatographic batch. In addition, incurred sample reanalysis was performed to confirm the reliability and reproducibility of the reported pomalidomide plasma concentrations.

All validation parameters were established and evaluated in accordance with the applicable guidelines for bioanalytical method validation [18,19,20,21].

#### 4.2.5. Statistical Analysis

The sample size was calculated based on standard bioequivalence assumptions, including a significance level of 5%, a statistical power of 80%, an acceptance range of 80.00–125.00%, and an expected intra-subject variability of approximately 26% [29]. An additional margin was included to account for potential dropouts and exclusions, ensuring the robustness of the statistical analysis. 

Pharmacokinetic parameters were obtained from plasma concentration–time profiles. Data analysis was performed using Phoenix^®^ WinNonlin^®^ version 8.5 (Certara, Radnor, PA, USA).

The area under the plasma concentration–time curve was calculated using the trapezoidal method, including the determination of AUC from time zero to the last measurable concentration (AUC_0–t_). The area under the concentration–time curve extrapolated to infinity (AUC_0–inf_) was also calculated and included in the descriptive pharmacokinetic analysis. The percentage of AUC extrapolated from the last measurable concentration to infinity (%AUCextrap) was evaluated for all subjects. 

The elimination half-life (t_1/2_) was calculated as ln(2)/Kel, where Kel was estimated from at least three points in the terminal log-linear phase, selected based on visual inspection and adjusted R^2^ criteria. 

The Cmax and Tmax values were read directly from the data without interpolation.

Analysis of variance (ANOVA) was conducted to assess bioequivalence with sequence, period, and treatment as fixed effects and subjects nested within sequence as a random effect.

The primary pharmacokinetic parameters for bioequivalence evaluation were Cmax and AUC_0–t_. Log-transformed data were used to estimate the 90% confidence intervals for the geometric mean ratios between the test and reference formulations. Bioequivalence was concluded when the confidence intervals for these ratios fell entirely within the predefined acceptance range of 80.00% to 125.00%, in accordance with regulatory requirements established by health authorities such as INVIMA (Colombia) and ISP (Chile) [18,19,20,21].

#### 4.2.6. Safety

All subjects were monitored throughout all phases of the study. Baseline and ongoing vital signs—including temperature, blood pressure, heart rate, and respiratory rate—were assessed continuously. Laboratory tests (hematology, urinalysis, and blood biochemistry), physical examinations, and electrocardiograms were performed at baseline and at the end of the study.

Adverse events were evaluated by the nursing and medical staff throughout the study. These events were graded as mild, moderate, or severe and classified as either suspected or not suspected to be causally related to the drug.

Subjects were instructed to promptly report any undesirable symptoms or medical conditions during the study or after the confinement period.

### 4.3. Biowaiver Approach for the Lower Strength

The biowaiver for the lower strength (3 mg) was conducted in accordance with international regulatory guidelines that allow the extrapolation of bioequivalence data from the highest strength (4 mg). This approach was based on the excipient proportionality, linear pharmacokinetics of pomalidomide, and comparable in vitro performance between the strengths [25].

The test product (Xetrane^®^ 3 mg pomalidomide hard capsules) and the comparator (bio-batch, Xetrane^®^ 4 mg pomalidomide hard capsules), which demonstrated in vivo bioequivalence to the reference product, were manufactured by the same company using identical manufacturing processes. Qualitative and quantitative proportionalities between the strengths were confirmed.

Comparative dissolution profiles were performed using twelve units of each formulation. The assay was conducted using USP Apparatus II (paddle) operated at 75 rpm, with the use of a sinker to prevent capsule flotation, at a controlled temperature of 37 ± 0.5 °C. The dissolution profiles were obtained in four media: quality control medium (0.1 N HCl), pH 1.2 (0.05 N HCl), pH 4.5 (acetate buffer), and pH 6.8 (phosphate buffer). 

The amount of pomalidomide dissolved at each sampling time was quantified using a validated HPLC-UV method. A similarity factor (f_2_) was used to evaluate dissolution profile similarity between the 3 mg and 4 mg strengths. The dissolution profiles were considered similar when f_2_ values were equal to or greater than 50.

## 5. Conclusions

This study successfully characterized the pharmacokinetic profile of pomalidomide for both the test and reference formulations, in compliance with applicable regulatory requirements. The two products (Xetrane^®^ and Imnovid^®^) showed similar pharmacokinetic parameters, with the 90% confidence intervals of the geometric mean ratios (test/reference) for log-transformed Cmax and AUC_0–t_ values fully contained within the predefined bioequivalence acceptance range of 80.00–125.00%. In addition, both formulations were well tolerated and exhibited similar safety profiles in healthy male subjects. These findings support the bioequivalence of the two formulations from a pharmacokinetic and regulatory perspective.

## Figures and Tables

**Table 1:** Demographic characteristics of study subjects.

Characteristic	Descriptive Statistics (*N* = 29)
Age (years)	
Mean_(±SD)_	25_(±5)_
Range	18–39
Weight (kg)	
Mean_(±SD)_	76_(±10)_
Range	59–92
Height (m)	
Mean_(±SD)_	1.72_(±0.06)_
Range	1.61–1.84
BMI (kg/m^2^)	
Mean_(±SD)_	25.31_(±2.91)_
Range	20.31–29.75

**Table 2:** Pharmacokinetic parameters of the pomalidomide hard capsule formulations (test and reference) in healthy subjects under fasting conditions (*N* = 29).

Parameter	Test	Reference
Cmax (ng/mL) Mean_±SD_	59.08_±12.07_	55.54_±11.10_
AUC_0–t_ (ng·h/mL) Mean_±SD_	487.08_±102.32_	515.72_±112.51_
AUC_0–inf_ (ng·h/mL) Mean_±SD_	510.67_±101.47_	539.07_±111.51_
Tmax (h) Mean _±SD_ Median (min–max)	1.93 (0.84) 2 (1–5)	2.44 (1.21) 2 (1–5)
Kel (1/h) Mean_±SD_	0.1131_±0.0209_	0.1115_±0.0185_
t_1/2_ (h) Mean_±SD_	6.35_±1.31_	6.40_±1.21_

Cmax: maximum plasma concentration; AUC_0–t_: area under the curve of plasma concentration vs. time from 0 to 48 h. AUC_0–inf_: area under the plasma concentration–time curve from time zero extrapolated to infinity; Tmax: time to reach Cmax; Kel: elimination constant; t_1/2_: elimination half-life.

**Table 3:** Geometric mean ratio and 90% confidence intervals from the bioequivalence analysis.

Parameter *	N	T/R Geometric Mean Ratio %	90% Confidence Interval	CV_ws_ %	Power %
Cmax	29	106.24	100.77–112.00	11.85	>99
AUC_0–t_	29	94.67	91.61–97.82	7.34	>99
AUC_0–inf_	29	94.63	92.12–97.82	6.72	>99

* Ln-transformed; CV_ws_, within-subject coefficient of variation.

**Table 4:** Non-serious adverse events reported during the bioequivalence study (*N* = 34 subjects; 12 non-serious adverse events reported).

Adverse Event ^1^	Number of Events (*n*)		Causality ^2^	Intensity
	Test	Reference		
Pruritus	4	3	Likely	Mild
Cutaneous eruption	2	2	Likely	Mild
Dental infection	1	0	Unlikely	Mild

^1^ Terminology based on MedDRA. ^2^ Based on WHO-UMC causality categories.

**Table 5:** Similarity factor (f_2_) values for comparative dissolution profiles between pomalidomide 3 mg and 4 mg hard capsule formulations.

Dissolution Medium	Similarity Factor (f_2_)	Conclusion
QC (0.1 N HCl)	59.5	Similar
pH 1.2 (0.05 N HCl)	54.4	Similar
pH 4.5 acetate buffer	56.2	Similar
pH 6.8 phosphate buffer	67.3	Similar

Similarity was concluded when f_2_ ≥ 50.

## Data Availability

The original contributions presented in this study are included in the article. Further inquiries can be directed to the corresponding authors.
